# Editorial: Role of Metabolism in Regulating Immune Cell Fate Decisions

**DOI:** 10.3389/fimmu.2020.00527

**Published:** 2020-03-24

**Authors:** Anna Ohradanova-Repic, Marianne Boes, Hannes Stockinger

**Affiliations:** ^1^Institute for Hygiene and Applied Immunology, Center for Pathophysiology, Infectiology and Immunology, Medical University of Vienna, Vienna, Austria; ^2^Department of Pediatrics and Center of Translational Immunology, University Medical Center Utrecht, Utrecht University, Utrecht, Netherlands

**Keywords:** immunometabolism, metabolic reprogramming, immune cell regulation, cell fate decisions, T cells, myeloid cells, innate lymphoid cells

Immunometabolism, an interplay between immunological and metabolic processes, describes not only the stepwise adaptation of intracellular metabolic pathways to sustain the bioenergetic demand of an immune response, but also how these metabolic adaptations directly affect immune cell functions and cell fate by controlling transcriptional, post-transcriptional and epigenetic events. The first concepts of immunometabolism date back to the 1930s, when Kempner and Peschel formulated their metabolic concepts of the physiology of inflammation using experiments they performed in a cantharidin-induced skin blister model [as described in Nagy and Haschemi (1)]. Immunometabolism was rediscovered in the twenty-first century, when on one hand, it had emerged that certain chronic, supposedly non-immune, pathologies including obesity contribute to mobilization of the immune system that drives metabolic abnormalities, leading to increased susceptibility to type 2 diabetes, cardiovascular and liver diseases, neurodegeneration and cancer. On the other hand, it was proposed that well-known cellular nutrient sensors, serine/theonine kinases AKT, APMK, LKB1, mTOR, and the transcriptional factor aryl hydrocarbon receptor (AhR) control a fate switch of T cells (2–5). The field has seen a tremendous development since then. This Research Topic contains 16 (Mini)Review, Opinion, and Original Research articles that review and expand our current understanding of the molecular underpinnings of immunological/metabolic cross-talk and metabolism-guided fate decisions of different immune cells during an immune response.

In one of the first articles of the Research Topic, Viola et al. provide a comprehensive overview of the main metabolic pathways in macrophages, and how these pathways are rewired to support the particular functions of the pro-inflammatory (M1) vs. anti-inflammatory (M2) macrophages. Special attention is given to the Krebs cycle metabolites citrate, itaconate and succinate, due to their non-metabolic roles in specific events during macrophage activation, and to disease-associated macrophage metabolic abnormalities. Wilson et al. continue to review the role of macrophage metabolic reprogramming and immune functions in the context of granulomatous diseases. Three examples of granulomatous disease are presented (tuberculosis, schistosomiasis, and sarcoidosis), with important similarities and differences critically discussed, highlighting dysregulated lipid metabolism as a common denominator in granulomatous disease progression. In their Mini Review, Sharif et al. focus on the role of the class I phosphoinositide-3-kinase (PI3K) signaling in sensing nutrients increased in obesity and subsequent rewiring of the metabolism and responses of adipose tissue macrophages, linking metabolically triggered inflammation (meta-inflammation) to insulin resistance and diabetes. Meta-inflammation is also studied in the Original Research article by Min et al. who demonstrate that pyruvate dehydrogenase kinase (PDK), which inhibits the pyruvate dehydrogenase-mediated conversion of cytosolic pyruvate to mitochondrial acetyl-CoA, is a metabolic checkpoint for polarization of macrophages to the M1 phenotype. Combined PDK2 and PDK4 deficiency (both global and hematopoietic cell-specific), or alternatively, pharmacological inhibition using a novel PDK inhibitor KPLH1130 prevents M1 macrophage polarization, reduces obesity-associated insulin resistance, and ameliorates adipose tissue inflammation, introducing a viable strategy for the treatment of inflammatory metabolic disorders. In the second Original Research article of this collection, Chapman et al. link proinflammatory stimulation of myeloid cells with ligands of the pattern recognition receptors Toll-like receptor 2 (TLR2) and nucleotide-binding oligomerization domain-containing protein 2 (NOD2) to metabolic rewiring needed for effector functions by identification of the deubiquitinating enzyme ataxin-3 downstream from TLR2 and NOD2, and demonstrate that ataxin-3 is necessary for optimal mitochondrial respiration and reactive oxygen species production, as well as for intracellular bacterial killing.

Given the particular importance of metabolic remodeling in regulation of T cell development, activation, function, differentiation, and survival, several Review articles within this collection tackle the metabolic control of T cell fate, each from a different angle. Konjar and Veldhoen thoroughly discuss recent insights in metabolic characteristics and phenotypes of CD8 T cell subsets, by side-by-side comparison of naive, circulating memory, effector and tissue resident CD8 T cells. Emphasis is given on tissue resident memory CD8 T cells at the epithelial barriers that show unique metabolic rewiring adapted to their niche in order to fulfill their roles—tissue homeostasis and immediate protection against microbial invasion. The review by Pacella and Piconese addresses the roles and regulation of cellular bioenergetic metabolic pathways in regulatory T cells (Treg) compared to conventional CD4 T cells. By critical analysis of metabolic and functional differences of the two cell types in metabolite-rich (liver, adipose tissue) vs. nutrient-restricted (tumor microenvironment) tissues, they highlight the higher capability of Tregs to adapt to metabolic hurdles, that could be explored for therapeutic purposes. Stark et al. review the metabolic requirements of Th2 cells during their early and late differentiation, focusing on the impact of glucose and lipid metabolism, mTOR activation, the nuclear receptor PPARγ and several extracellular metabolites that directly promote Th2 functions, as well as on metabolic interventions targeting type 2 inflammation. Colamatteo et al. provide a comprehensive review of the role of microRNAs (miRNAs) in regulating T cell metabolism and how the dysregulation of this control can lead to autoimmunity. They also speculate on the possibility that the interplay between miRNAs and metabolism in T cells may help identifying novel miRNA-based therapeutic strategies to treat effector T cell immunometabolic alterations in autoimmune and chronic inflammatory diseases. In their Opinion article, Mondanelli et al. discuss the immunoregulatory interplay between arginine and tryptophan metabolism, connecting deregulated expression of the catabolising enzymes for these amino acids [arginase 1 (ARG1) or indoleamine 2,3-dioxygenase 1 (IDO1), respectively] in neoplasia and autoimmune diseases to functional reprogramming of immune cells, dendritic cells, and T cells in particular. At the cellular level, Audrito et al. give a comprehensive overview on the crosstalk between tumor cells, stromal cells, and infiltrating immune cells (tumor infiltrating lymphocytes, tumor-associated macrophages and neutrophils, myeloid-derived suppressor cells) in tumor microenvironment with specific focus on nicotinamide adenine dinucleotide (NAD) metabolism. The role of the entire “NADome” (NAD metabolites, NAD-biosynthetic and -consuming enzymes) in cancer growth and immune evasion and currently pursuit therapeutic strategies for NADome blockade are critically reviewed.

In a Mini Review, Yerinde et al. discuss a crosstalk between the metabolic and the epigenetic regulation of CD8 T cell differentiation and function and also briefly summarize how metabolic signals from the tumor microenvironment (or virus-infected cells) shape the epigenetic landscape of CD8 T cells. Similarly, Magalhaes et al. draw parallels between mechanisms employed by tumor cells and viruses to influence T cell metabolic regulation and propose an emerging view that metabolic changes in tumors and virally infected cells uniformly create a suppressive microenvironment leading to inhibition of effector CD4 and CD8 T cells. Therefore, these reviews provide an important insight into mechanisms that underlie T cell exhaustion in anti-tumor and anti-viral immunity, which could inspire development of effective therapeutic interventions against it. This view is well-complemented by the Review article of Mayer et al. detailing crucial cellular metabolic pathways that are being utilized by several DNA and RNA viruses for their replication and survival. The dichotomy between the strategy for host cell manipulation—DNA viruses preferentially employ the transcriptional control of key metabolic pathways, while RNA viruses rely on post-transcriptional modifications—is noted and in this view, currently pursuit strategies for metabolism-targeting interventions against different viruses are clearly summarized.

Innate lymphoid cells (ILCs), are relatively recently discovered lymphocytes lacking diversified adaptive antigen receptors. These largely tissue-resident cells play an important role in tissue homeostasis, host defense at mucosal barriers and tissue repair (6). There is increasing evidence linking ILCs with metabolic homeostasis, and immunometabolic regulation of ILCs is an emerging frontier. At a cellular level, Poznanski and Ashkar critically review recent literature to answer the question whether metabolism—or phenotype—can define the functional fate of human natural killer (NK) cells, and provide evidence that indeed differences in metabolism (especially in glucose metabolism) better discriminate between cytotoxic, regulatory and memory NK cells than surface markers. At the whole organ level, Willinger takes a systemic approach, and in his Review thoroughly discusses metabolic signals that regulate ILC homing to the particular tissues and their strategic positioning in healthy and inflamed tissues. Trafficking of both ILC precursors and mature ILCs (NK cells, ILC1s, ILC2s, ILC3s, and lymphoid tissue-inducer, LTi cells), including species-specific differences between humans and mice, are comprehensively covered.

In conclusion, this collection of Review and Original Research articles critically summarizes current understanding of intertwining of metabolic and signaling pathways as ways to determine immune cell fates and ultimately, the ensuing immune response. We anticipate that the articles in the present collection will serve as an inspiration for future research, that will lead to deeper knowledge of cell-intrinsic and -extrinsic metabolic cues regulating cell fate decisions in different immune cell types, and subsequently to a development of novel medicines for immune-mediated diseases.

## Author Contributions

All authors listed have made a substantial, direct and intellectual contribution to the work, and approved it for publication.

### Conflict of Interest

The authors declare that the research was conducted in the absence of any commercial or financial relationships that could be construed as a potential conflict of interest.

## References

[1] Nagy C Haschemi A. Time and demand are two critical dimensions of immunometabolism: the process of macrophage activation and the pentose phosphate pathway. Front Immunol. (2015) 6:164. 10.3389/fimmu.2015.00164 25904920 PMC4389563

[2] Mathis D Shoelson SE. Immunometabolism: an emerging frontier. Nat Rev Immunol. (2011) 11:81. 10.1038/nri2922 21469396 PMC4784680

[3] Hotamisligil GS. Foundations of immunometabolism and implications for metabolic health and disease. Immunity. (2017) 47:406–20. 10.1016/j.immuni.2017.08.009 28930657 PMC5627521

[4] Finlay D Cantrell DA. Metabolism, migration and memory in cytotoxic T cells. Nat Rev Immunol. (2011) 11:109–17. 10.1038/nri2888 21233853 PMC3521506

[5] Mezrich JD Fechner JH Zhang X Johnson BP Burlingham WJ Bradfield CA. An interaction between kynurenine and the aryl hydrocarbon receptor can generate regulatory T cells. J Immunol. (2010) 185:3190–8. 10.4049/jimmunol.0903670 20720200 PMC2952546

[6] Vivier E Artis D Colonna M Diefenbach A Di Santo JP Eberl G. Innate lymphoid cells: 10 years on. Cell. (2018) 174:1054–66. 10.1016/j.cell.2018.07.017 30142344

